# Genetic architecture and evolution of stripe rust resistance uncovered using diverse panels of wheat lines and North American *Puccinia striiformis* f. sp. *tritici* isolates

**DOI:** 10.1007/s00122-026-05363-x

**Published:** 2026-09-08

**Authors:** Buket Sahin, Meinan Wang, Jason D. Zurn, Xiaoting Xu, Guihua Bai, Alina Akhunova, Xianming Chen, Eduard Akhunov

**Affiliations:** 1 Department of Plant Pathology, Kansas State University, Manhattan, USA; 2 Wheat Genetics Resource Center, Kansas State University, Manhattan, USA; 3 Department of Plant Pathology, Washington State University, Pullman, WA USA; 4 Department of Agronomy, Kansas State University, Manhattan, USA; 5 Integrated Genomics Facility, Kansas State University, Manhattan, USA; 6 USDA ARS, Wheat Health, Genetics and Quality Research Unit, Pullman, WA USA

## Abstract

**Key message:**

Screening global wheat germplasm with a diverse collection of pathogen races expands the catalog of novel Yr loci and identifies new sources of broad-spectrum resistance against evolving Pst populations.

**Abstract:**

Newly emerging highly virulent races of *Puccinia striiformis* f. sp. *tritici* (*Pst*) often defeat deployed resistance genes (*Yr*), highlighting the need for novel sources of durable resistance. A global diversity panel of 377 spring wheat (*Triticum aestivum* L.) lines was screened for all-stage resistance (ASR) against a panel of diverse 20 *Pst* isolates at the seedling stage and for adult-plant-stage resistance (APR) against natural mix of field races. Genome-wide association mapping identified 77 unique *Yr* loci. Of these, 34 overlapped with the previously mapped 1150 *Yr* loci, confirming the robustness of our GWAS results, while 43 were likely novel. Comparison of the nine adult-plant-stage *Yr* loci mapped in our study with known APR genes identified only one overlap, with *Yr29*. Except for *Yr29*, APR genes *Yr18* and *Yr36* were detected at low frequencies, indicating that resistance in our panel may arise from less characterized or novel sources. Two wheat lines, lacking widely effective *Yr5* and *Yr15* alleles, exhibited resistance to all 20 *Pst* races at the seedling stage and natural field races at the adult stage, suggesting that they may carry novel, broad-spectrum ASR alleles. Wheat improvement had no effect on the frequency of ASR alleles but resulted in a threefold increase in the frequency of APR alleles, suggesting that the latter were subjected to more consistent breeding selection over time. Our findings underscore the value of combined screening of diverse germplasm with diverse pathogen races to identify novel sources of broad-spectrum resistance for breeding stripe rust resistant cultivars.

**Supplementary Information:**

The online version contains supplementary material available at https://doi.org/10.1007/s00122-026-05363-x.

## Introduction

Wheat (*Triticum aestivum* L.) is a cornerstone of global agriculture, cultivated on more than 220 million hectares worldwide and providing a primary source of food and nutrition for billions of people (FAOSTAT 2023). Wheat yields are consistently challenged by a variety of biotic and abiotic stress factors. Among biotic constraints, stripe rust caused by *Puccinia striiformis* f. sp. *tritici* (*Pst*) remains one of the most widespread and devastating fungal diseases affecting wheat. Over the past two decades, *Pst* races have emerged that are more virulent or adapted to warmer environments, enabling the pathogen to spread into regions previously considered unsuitable for disease development (Chen 2005). These changes have led to more frequent and severe epidemics, with annual yield losses estimated to exceed one billion US dollars (Chen 2020). The scale and persistence of these losses highlight the urgent need for cultivars that offer durable and effective resistance to evolving *Pst* populations (Beddow et al. 2015).

Genetic resistance is the most sustainable method for controlling stripe rust in wheat. Resistance is classified into two categories: all-stage resistance (ASR), which is usually race-specific and controlled by single major genes, and adult-plant resistance (APR), which is governed by allelic variants of genes with small effects, providing partial but more durable protection (Singh et al. 2015; Huerta-Espino et al. 2020; Wang et al. 2023). ASR is active across all growth stages but is often overcome by new pathogen races (Ballini et al. 2013; Singh et al. 2015; Schwessinger 2016), particularly when deployed as single-gene resistance. To extend its efficacy, ASR is commonly incorporated as part of multi-gene combinations (Bulli et al. 2016; Zhang et al. 2019). In contrast, APR tends to be more stable over time due to its complex genetic basis, lower race specificity, and lower levels of resistance, which reduces selection pressure on pathogen populations (Chen 2013; Brown 2015; Singh et al. 2016). To date, 87 named *Yr* genes have been included into the wheat gene catalogue, of which 18 confer APR and the remaining provide ASR (McIntosh 2024). Modern wheat breeding programs have historically relied on elite germplasm as primary sources of resistance, largely due to their agronomic performance and adaptation (Reif et al. 2005; Lopes et al. 2015). These elite varieties carry a few multi-gene combinations selected for their ability to provide resistance to endemic *Pst* races. The effectiveness of many resistance loci in commercial varieties has declined in some regions due to the ongoing evolution of *Pst* populations (Hovmøller et al. 2016; Riella et al. 2024), including recent worrying reports of *Yr15* breakdown in Europe (Davis et al. 2025) This underscores the need to discover and utilize additional resistance sources that remain effective against newly emerging *Pst* races. Beyond elite germplasm, a wide range of genetic resources, including landraces, historical cultivars, and breeding lines, offer valuable reservoirs of disease resistance that have received limited attention in modern breeding efforts. Many of these materials were selected under local environmental pressures, allowing them to co-evolve with diverse *Pst* populations and retain greater allelic diversity (Zuev et al. 2024). In contrast to elite lines shaped by repeated selection and reduced genetic variation, older accessions often carry rare and previously untapped resistance alleles (Adhikari et al. 2012). Landraces from regions such as Turkey, Ethiopia, and Central Asia have shown both ASR and APR responses to stripe rust, underscoring their potential value in resistance breeding programs (Zegeye et al. 2014; Sehgal et al. 2016; Jia et al. 2020). However, a large proportion of accessions conserved in global gene banks remain uncharacterized for resistance to currently prevalent *Pst* races, leaving much of their genetic potential unexplored. Systematic evaluation of these underutilized resources is critical for identifying novel resistance loci and expanding the genetic base of wheat improvement.

Genome-wide association studies (GWAS) have proven to be an effective approach for dissecting the genetic architecture of complex traits, such as disease resistance, particularly when applied to germplasm collections encompassing broad natural variation (Yu et al. 2006). In wheat, GWAS has been successfully employed to detect numerous loci associated with stripe rust resistance, particularly within elite breeding materials (Kertho et al. 2015; Bulli et al. 2016; Liu et al. 2020; Gao et al. 2024; Wu et al. 2025). Nevertheless, studies incorporating genetically diverse and globally representative germplasm panels remain limited, and the extent of novel resistance variation segregating in unimproved materials is still insufficiently characterized. Expanding GWAS efforts to include these underutilized genetic resources holds substantial promise for the discovery of novel resistance loci and for enhancing the long-term efficacy of resistance breeding strategies.

Building on this premise, the present study evaluated a diverse panel of 377 spring wheat accessions from the USDA National Small Grains Collection. This panel includes landraces, historical cultivars, breeding lines, and other genetic materials representing over a century of global wheat diversity and encompassing major centers of origin, diversification, and adaptation. The primary objective was to identify genomic regions associated with resistance to stripe rust by applying genome-wide association mapping to this genetically diverse panel, with the goal of capturing both common and rare alleles that may have been beyond the resolution of prior studies focused on narrower populations. In parallel, the study aimed to dissect the genetic architecture of resistance by estimating the contribution of individual loci to phenotypic variation and by identifying genomic regions conferring consistent resistance across 20 distinct *Pst* races. This comparative, multi-race approach enables the differentiation of loci associated with ASR from those contributing to APR, supporting the prioritization of loci with stable effects across environments and pathotypes.

## Materials and methods

### Plant materials and genotyping

A genetically diverse set of 377 spring wheat accessions was selected from the USDA National Small Grains Collection to represent broad allelic variation within global wheat diversity (Table S1). The accessions include landraces, historical cultivars, breeding selections, and other genetic materials originating from 79 countries across seven geographic regions, with collection years spanning from 1904 to 2017.

Genotyping was performed as described by He et al. (2022), using a combined platform approach that integrated the 90K iSelect SNP array, genotyping by sequencing (GBS) based on restriction enzyme digestion, targeted resequencing of regulatory regions, and RNA-seq-based SNP discovery. This multi-tiered strategy provided high-density, genome-wide coverage suitable for high-resolution association analysis. Missing genotype calls were imputed with Beagle, incorporating the complete marker set (Browning et al. 2021). Quality control filtering excluded SNPs with a minor allele frequency (MAF) below 0.05, a heterozygosity rate above 3%, or missing data exceeding 50% at the marker or accession level. After filtering, a total of 1,521,858 high-confidence SNPs mapped to CS v1.0 reference were retained for downstream analyses. The variants are available from the EMBL EVA database under accession PRJNA1480388.

In addition to genome-wide SNP genotyping, the panel was genotyped using five KASP (Kompetitive Allele-Specific Polymerase Chain Reaction) markers linked to previously characterized broadly effective *Yr* resistance genes. Genotyping was performed using markers available at the USDA-ARS Genotyping Laboratory in Manhattan, KS, targeting genes *Yr5* (Marchal et al. 2018) and *Yr15* (Klymiuk et al. 2018), which confer broad-spectrum ASR, and *Yr36* (Rasheed et al. 2016), *Lr34/Yr18* (Lagudah et al. 2009), and *Lr46/Yr29*, which are associated with pleiotropic broad-spectrum APR.

### Seedling stage evaluations

Resistance to stripe rust was evaluated at the seedling stage under controlled greenhouse conditions in 2021 and 2022 using 20 *Pst* races with distinct virulence profiles (Table 1). These races were selected to represent a range of virulence combinations and included both well-established and recently identified pathotypes. Each race was phenotyped using a standard set of differential wheat lines carrying single *Yr* resistance genes (Wan and Chen 2014), and their virulence patterns were validated against reference isolates to ensure consistency across trials. Growing plants and inoculation were conducted following the standard procedure and conditions (Chen and Wang 2025). Briefly, 5–7 seeds of each line were planted in a pot of 6 × 6 × 6 cm filled with soil mixture containing 6 L peat moss, 2 L perlite, 3 L sand, 3 L Palouse silt loam soil, 4 L vermiculite, 250 g Osmocote 14-14-14 and 2 L water premixed in a soil mixer rotating for 30 min. The pots were placed in a greenhouse set at 10–25 °C with a daily 16 h photoperiod for growing seedlings for 10–12 days. At the two-leaf stage, seedlings were uniformly inoculated with a suspension of fresh urediniospores (spores of just harvested or dried spores stored at 4 °C less than two months) in Novec 7100 Engineered Fluid (3M, St. Paul, MN, USA) at the rate of 5 mg/mL using a spray inoculator. Inoculated plants were kept in a dew chamber at 10 °C for 24 h in dark and then placed in a growth chamber set at a diurnal temperature cycle 4–20 °C and a corresponding 8/16 h dark/light cycle.

**Table 1:** *Puccinia striiformis* f. sp. *tritici* races used in seedling tests, including isolates and virulence formulae

Race	Isolate	Virulence formula*
PSTv-1	19WA-32	1
PSTv-4	20WA-19-1	1, 6, 9, 17, 27, SP, 76
PSTv-11	09-134	1, 6, 7, 8, 9, 17, 27, 43, 44, Exp2, 76
PSTv-14	17-326	1, 6, 7, 8, 9, 17, 27, 43, 44, 85, Exp2, 76
PSTv-17	21KS-2-1	1, 6, 7, 8, 9, 17, 27, 43, 44, SP, Exp2, 76
PSTv-18	11-281	none
PSTv-20	93TX-210	17
PSTv-21	90AR-1	6, 17
PSTv-27	93-27-Yr10	10, 17, 24, 32, 85
PSTv-32	21K-41-Yr9	6, 7, 8, 9, 44, 85, Exp2
PSTv-37	21WA-112	6, 7, 8, 9, 17, 27, 43, 44, 85, Exp2
PSTv-44	21WA-145-Yr1	1, 7, 8, 9, 27, 44, Exp2, 76
PSTv-46	21WA-141	1, 6, 9, 17, 27, 76
PSTv-48	21WA-70-Yr1	1, 6, 9, 76
PSTv-52	22LA-5	6, 7, 8, 9, 17, 27, 43, 44, Exp2
PSTv-64	06-19NB	6, 9
PSTv-119	20WA-106	6, 7, 8, 17, 27, 43, 44, Exp2
PSTv-120	20WA-189-Yr1	1, 6, 9, 27, SP, 76
PSTv-220	20CA-29	1, 6, 7, 8, 9, 17, 27, 43, 44, SP, 85, Exp2, 76
PSTv-326	20WA-172-2	1, 6, 7, 8, 9, 10, 17, 24, 27, 32, 43, 44, 85, Exp2

^*^Infection types (0–6 = avirulent, 7–9 = virulent); virulence formula lists *Yr* genes to which the *Pst* race is virulent based on their scores on the 18 genes (*Yr1*, *Yr5*, *Yr6*, *Yr7*, *Yr8*, *Yr9*, *Yr10*, *Yr15*, *Yr17*, *Yr24*, *Yr27*, *Yr32*, *Yr43*, *Yr44*, *YrSP*, *Yr85* (= *YrTr1*), *YrExp2,* and *Yr76* (= *YrTye*) in the *Yr* single-gene differentials (Wan and Chen 2014; Wang et al. 2022).

Stripe rust responses were assessed using the 0–9 infection-type (IT) scale previously described for stripe rust (Line and Qayoum 1992). IT scores of 0–3 indicated resistant reactions, typically showing no visible symptom or necrosis and chlorosis with no to little sporulation. Scores of 4–6 were classified as intermediate, involving moderate sporulation on chlorotic or necrotic tissues, while scores of 7–9 corresponding to susceptible response, characterized by heavy sporulation with or without visible necrosis or chlorosis.

### Adult-plant phenotyping for stripe rust response

Adult-plant phenotyping for responses to *Pst* was conducted under either natural infection or artificial inoculation conditions during the 2022 and 2023 growing seasons. The spring wheat panel was planted at three locations in Washington State–Pullman, Mount Vernon, and Central Ferry–in 2022 and 2023. The Pullman and Central Ferry sites are in eastern Washington about 50 kilometers apart, and Mount Vernon is in western Washington and about 500 kilometers from Pullman and Central Ferry. The Pullman and Mount Vernon fields are rainfed, and the Central Ferry field is under limited irrigation. The eastern and western Washington sites vary in wheat stripe rust seasons and *Pst* race compositions due to different environments. The Pst race composition at each phenotyping location is presented in Table S2.

In each year, the spring wheat panel was planted together with other spring wheat nurseries in April at the three locations. The 377 wheat accessions were planted in 50 cm rows with 20 cm between rows with 5-gram seeds in a single row for each line. The susceptible check AvS was planted every 20 rows and surrounding the entire spring wheat experimental field to create uniform stripe rust pressure. The fields were maintained with standard management practices for each region. In both 2022 and 2023, the spring wheat field at Pullman was indirectly inoculated as the adjacent winter wheat field was inoculated in late April with *Pst* urediniospores suspended in Novec 7100 as described above. The spores used for field inoculation were collected from the same location in 2021 and 2022, respectively, and stored in liquid nitrogen. The collected isolates were pathotyped using the differential set of lines to determine their race types prior to inoculating for the 2022 and 2023 field seasons. The survey of field race composition conducted in 2022 and 2023 showed that each location contained a complex mixture of *Pst* isolates, with a few dominant races occurring at high frequency (Table S2). In 2022, PSTv-37 was the most prevalent race in WA, accounting for approximately 41% of all isolates, followed by PSTv-36 (11%), PSTv-18 (8.5%), and PSTv-39 (7.3%). Several additional races such as PSTv-40, PSTv-41, PSTv-52, PSTv-198, and PSTv-201 were each detected at frequencies between 3 and 5%, while the remaining races occurred at ≤1%. In 2023, PSTv-37 remained dominant in WA, representing about 61% of all isolates, with PSTv-47 and PSTv-41 (each 7.3%) and PSTv-35 (5.2%) also detected at moderate frequency. All other races were found at ≤1%. Across both years, 13 of the 20 races evaluated in seedling tests were also detected in the field (PSTv-4, PSTv-11, PSTv-14, PSTv-18, PSTv-32, PSTv-37, PSTv-46, PSTv-48, PSTv-52, PSTv-64, PSTv-119, PSTv-120, and PSTv-220), indicating substantial race overlap between the greenhouse seedling tests and field tests. Race composition data were obtained from the Annual Stripe Rust Race Data Reports (https://striperust.wsu.edu/races/data/) (WSU 2022).

Stripe rust response was visually scored as IT 0–9 as described above, and severity (Sev) was scored using a modified Cobb scale to estimate the percentage of infected leaf area (Peterson et al. 1948). The Mount Vernon field was rated twice at the early jointing stage and the flowering stage, while the Pullman and Central Ferry fields were rated at the flowering to milk stages. Only the stripe rust data of the flowering to milk stage were used in this study to represent the adult-plant response for comparison with the seedling response from the greenhouse tests. Because the stripe rust pressure at Central Ferry was too low to allow reliable data collection in 2023, this location-year environment was discarded. In total, we obtained stripe rust data from five location-year environments that were used for this study.

To obtain a composite measure of resistance, the coefficient of infection (CI) was calculated by multiplying disease severity (%) by the IT score and dividing the result by 10, following the previously described method (Saari and Wilcoxson 2003). This scaling normalizes CI values within a 0–90 range, enabling more meaningful comparisons across genotypes and environments.

### PCA, linkage disequilibrium (LD) and kinship analyses

Principal component analysis (PCA) was performed to assess population structure among the 377 spring wheat accessions. The analysis was conducted using filtered and imputed SNP markers with the “prcomp” function in R (version 4.2.1). The genotype matrix was mean-centered and scaled prior to computation. The proportion of variance explained by each principal component (PC) was calculated by dividing the eigenvalues for each PC by the sum of all PC eigenvalues. Pairwise scatterplots using the first three PCs were generated to visualize clustering and potential subpopulation patterns.

LD was calculated as the squared allele frequency correlation (*r*2) between SNP pairs using PLINK v1.9 (Purcell et al. 2007), following the method described by (Hill and Robertson 1968). LD decay was analyzed separately for the A, B, and D subgenomes, as well as across the whole genome. Mean *r*2 values were computed for increasing physical distance bins, and LD decay curves were fitted using locally weighted scatterplot smoothing (LOESS) (Cleveland 1979) implemented in the “ggplot2” package. The physical distance at which *r*2 dropped below 0.2 was used to define the extent of LD decay.

A genomic relationship matrix (GRM) was computed to estimate pairwise kinship coefficients among accessions. The GRM was calculated using the “A.mat” function in the “sommer” package (Covarrubias-Pazaran 2016), following the previously described method (VanRaden 2008).

### Genome-wide association mapping

Association analyses were conducted using the multi-locus mixed model (MLMM) framework as implemented in the GAPIT v3.3 package in R (Segura et al. 2012; Wang and Zhang 2021). The MLMM model iteratively fits fixed effects of significant markers while simultaneously modeling background genetic relatedness as a random effect using GRM (VanRaden 2008). Population structure was corrected by incorporating PCs derived from PCA on the genotype matrix. The number of PCs in the model was optimized separately for each phenotype by visually inspecting quantile–quantile (Q–Q) plots. The final PC number was chosen as the lowest number of PCs that reduced systematic deviation from the expected null distribution while avoiding apparent overcorrection of the association signal. Genomic inflation was evaluated using the genomic control inflation factor (*λ*GC), and the selected PC number, *λ*GC value, and corresponding Q-Q plot were reported for each analysis in Table S3 and Figure S1.

Significance thresholds for marker–trait associations were determined using the false discovery rate (FDR) (Benjamini and Hochberg 1995), with a cutoff of FDR ≤ 0.05. Non-redundant lead SNPs for each trait were identified by grouping all significant marker-trait associations based on LD threshold of *r*2 ≥ 0.6 in a 10 Mb window. Following model fitting, results were summarized across races to identify both race-specific and overlapping resistance loci. Regions with multiple significant SNPs not overlapping with the ASR loci were considered as potential APR loci.

To facilitate the visualization of genome-wide marker–trait associations for each race, Manhattan plots were generated using the “ggplot2” R package (Wickham 2016), highlighting the chromosomal distribution and statistical significance of SNPs across the wheat genome. Peaks corresponding to significant associations were further examined for their proximity to known resistance loci and for potential pleiotropic effects across races.

### LD with known *Yr* genes and QTLs

To detect overlap between GWAS-identified loci and previously reported stripe rust resistance regions, a targeted linkage disequilibrium (LD) analysis was conducted using a comprehensive reference set of 1,150 known loci. This set combined previously curated *354* Yr genes and QTLs (Tong et al. 2024), originally mapped to RefSeq v2.1 (Zhu et al. 2021), and 790 additional resistance loci reported by Wu et al. (2025), which were already aligned to the IWGSC RefSeq v1.0 reference genome (The International Wheat Genome Sequencing Consortium (IWGSC) 2018). We have also added information about six APR loci missing in these two datasets: *Yr39*, *Yr55*, *Yr58*, *Yr59*, *Yr80*, and *Yr86* (Chhetri et al. 2016; Nsabiyera et al. 2018; Li et al. 2022; Zheng et al. 2022; Cao et al. 2024; Du et al. 2025). To ensure coordinate compatibility with our SNP dataset, physical positions from Tong et al. (2024) were converted to RefSeq v1.0 coordinates, while no conversion was required for the loci from Wu et al. (2025). Because physical positions of previously reported markers and QTLs were obtained from studies that may have already involved projection or coordinate conversion across reference assemblies, the comparison with known *Yr* loci was treated as an approximate guide rather than exact positional alignment. For each known resistance locus, a ±10 Mb window was defined, and all SNPs within these intervals were extracted from the filtered genotype matrix. Pairwise LD (*r*2) was calculated between significant GWAS SNPs and the SNPs located within these predefined windows using PLINK v1.9 (Purcell et al. 2007). SNP pairs with *r*2 ≥ 0.6 were considered as potentially co-localized. Loci meeting this threshold were classified as previously reported or known, while those showing no evidence of overlap or LD with reference loci were retained as putatively novel associations.

## Results

### Seedling responses to stripe rust

**Fig. 1:**
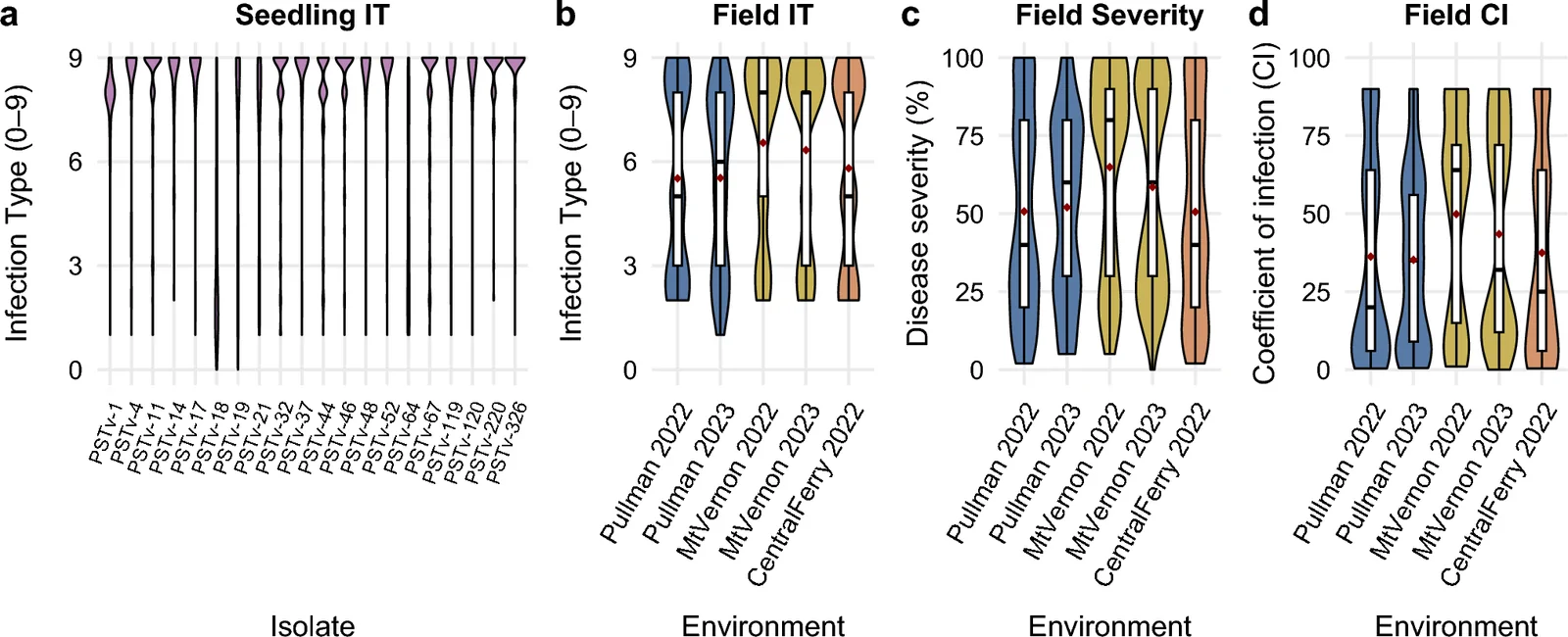
Distribution of stripe rust response scores in the diverse wheat panel. **a** Violin density plots show the distribution of seedling infection-type (IT) scores collected for 377 wheat lines under controlled greenhouse conditions. **b–d** Violin density plots with embedded boxplots show the distribution of IT scores (**b**), disease severity scores **c**, and coefficient of infection (CI) **d** evaluated at the adult-plant stage in field trials across three locations in 2022 (Pullman, Mount Vernon, and Central Ferry) and two locations in 2023 (Pullman and Mount Vernon)

The 377 spring wheat accessions displayed a wide range of responses to infection with 20 *Pst* races in the seedling stage, with IT scores ranging from 0 (immune) to 9 (fully susceptible) (Fig. 1a, Table S4). Based on a resistance threshold of IT ≤ 6, only two races were avirulent in more than 65% of accessions—PSTv-18 (83.02%) and PSTv-64 (68.97%). For the majority of the races (18 out of 20), over 50% of accessions showed susceptible reactions (IT ≥ 7), indicating the rarity of effective ASR alleles within the panel. The most virulent races were PSTv-37 and PSTv-326, for which only 2.65% and 1.86% of accessions, respectively, exhibited resistance. Overall, the panel exhibited race-specific variation, with substantial differences in resistance frequencies across races.

A total of 50 accessions (13.3%) were fully susceptible, exhibiting IT scores ≥ 7 against every race tested (Table S4). The remaining accessions displayed variable responses across isolates, indicative of the presence of race-specific resistance genes with intermediate to major effects on disease progression. Eleven spring wheat lines from the diversity panel exhibited resistance to at least 75% of the 20 *Pst* races evaluated at the seedling stage (Table S4). Notably, lines such as GF104 (CItr 15089), GF161 (PI 189794), GF225 (PI 207115), GF231 (PI 366981), and GF257 (PI 137758) showed resistance to 18 or more of the 20 *Pst* races, including highly virulent pathotypes PSTv-37, PSTv-52, PSTv-220, and PSTv-326. Among them, GF104 and GF225 were resistant to all 20 races and also remained resistant at the adult-plant stage. These genotypes represent valuable sources of ASR for breeding programs focused on enhancing durable and broad-spectrum resistance to stripe rust. These findings highlight the diversity of ASR genes within the panel and emphasize the importance of screening diverse germplasm with diverse pathogen races to identify rare accessions with broad-spectrum responses for use in resistance breeding programs.

### Adult-plant responses to stripe rust

The stripe rust pressure from either natural infection (Central Ferry and Mount Vernon) or artificial inoculation (Pullman) was high across all five environments, as shown by the consistent IT 9 and 100% severity of the susceptible check “Avocet S” in the five location-year environments (Fig. 1b, Tables S5 and S6). The uniformity of “Avocet S” responses across the location-year environments demonstrates that the stripe rust phenotype data were suitable for GWAS analysis. Each trial (location-year combination) was analyzed independently for IT, Sev, and CI, providing insights into the genetic basis of and environmental effect on ASR and/or APR observed at the adult-plant stage in the fields. Pearson correlation analyses across field trials revealed moderate-to-high consistency in disease response traits (Figure S2). The strongest correlations (Pearson’s *r* ≈ 0.7) were observed between the 2022 and 2023 trials at both Pullman and Mount Vernon for all three phenotypes (IT, Sev, and CI), indicating that lines in the panel exhibited consistent field resistance within each location across years.

In 2022, mean IT values ranged from 5.52 ± 2.71 in Pullman to 6.54 ± 2.55 in Mount Vernon, while CI ranged from 36.23 ± 32.36 to 49.88 ± 32.33, respectively (Table S5). Central Ferry, evaluated only in 2022, had an intermediate IT profile (IT = 5.81 ± 2.69; CI = 37.47 ± 31.97), with fewer highly susceptible lines. In 2023, stripe rust pressure remained stable, with Pullman and Mount Vernon showing mean ITs of 5.53 ± 2.51 and 6.33 ± 2.66, respectively (Table S7). IT score distributions (Fig. 1b) further illustrated these differences: Pullman displayed broader phenotypic variability, indicating greater genotypic diversity in resistance levels, while Mount Vernon exhibited more compressed distributions, with a larger proportion of susceptible responses. The percentage of accessions classified as resistant (IT ≤ 3) ranged from 22.7% (Mount Vernon 2022) to 29.6% (Central Ferry 2022) and increased slightly in 2023 to 26.8% (Mount Vernon) and 29.3% (Pullman), highlighting both spatial and temporal variation in adult-plant resistance expression.

Severity and CI distributions (Fig. 1c and d) followed a similar pattern: Pullman showed a wider range of values and a larger proportion of moderately resistant lines, while Mount Vernon exhibited higher mean severity and CI scores with more uniform, susceptible responses. The higher mean severity and CI values observed at Mount Vernon likely reflect the local race composition, which was dominated by highly virulent races such as PSTv-37, along with PSTv-36, PSTv-18, and PSTv-39 in 2022. Similarly, in 2023, PSTv-37 remained predominant, accompanied by PSTv-47, PSTv-41, and PSTv-35. The prevalence of these aggressive races likely contributed to the higher mean IT, Sev, and CI values and the reduced variability observed at this location.

Among the tested lines, 84 were resistant (IT ≤ 3) or intermediately resistant (IT ≤ 6) in at least one field environment. Of these, 40 accessions were classified as highly resistant in at least one trial (Tables S5–S7). Notably, GF75 (PI 351907) and GF358 (PI 272377), which displayed complete susceptibility at the seedling stage (IT ≥ 7 to all 20 *Pst* isolates), exhibited consistent adult-plant resistance across both years, with field IT scores reduced to 0–3 and lower disease severities, suggesting the presence of non-race-specific APR. Conversely, GF104 (CItr 15089) and GF225 (PI 207115) showed resistance to all races in the seedling tests and resistant/intermediate resistant responses across most field environments, indicative of broad-spectrum ASR.

### GWAS for seedling stripe rust responses

For GWAS, 625,579 markers were assigned to the A genome, 773,265 to the B genome, and 123,014 to the D genome. Principal component analysis showed that PC1, PC2, and PC3 explained 9.06, 6.24, and 3.47% of the total genetic variance, respectively, with the first ten components cumulatively accounting for 30.98% of the variation. This moderate level of population structure suggests the lack of strong underlying genetic stratification within the panel. Genome-wide LD analysis revealed variation in LD decay rates across subgenomes, with *r*2 values dropping below 0.2 around 950–1000 kb in the A and B genomes and within 1,300 kb in the *D* genome. These results suggest that the diversity panel provides sufficiently high resolution for locus mapping at the megabase level.

**Fig. 2:**
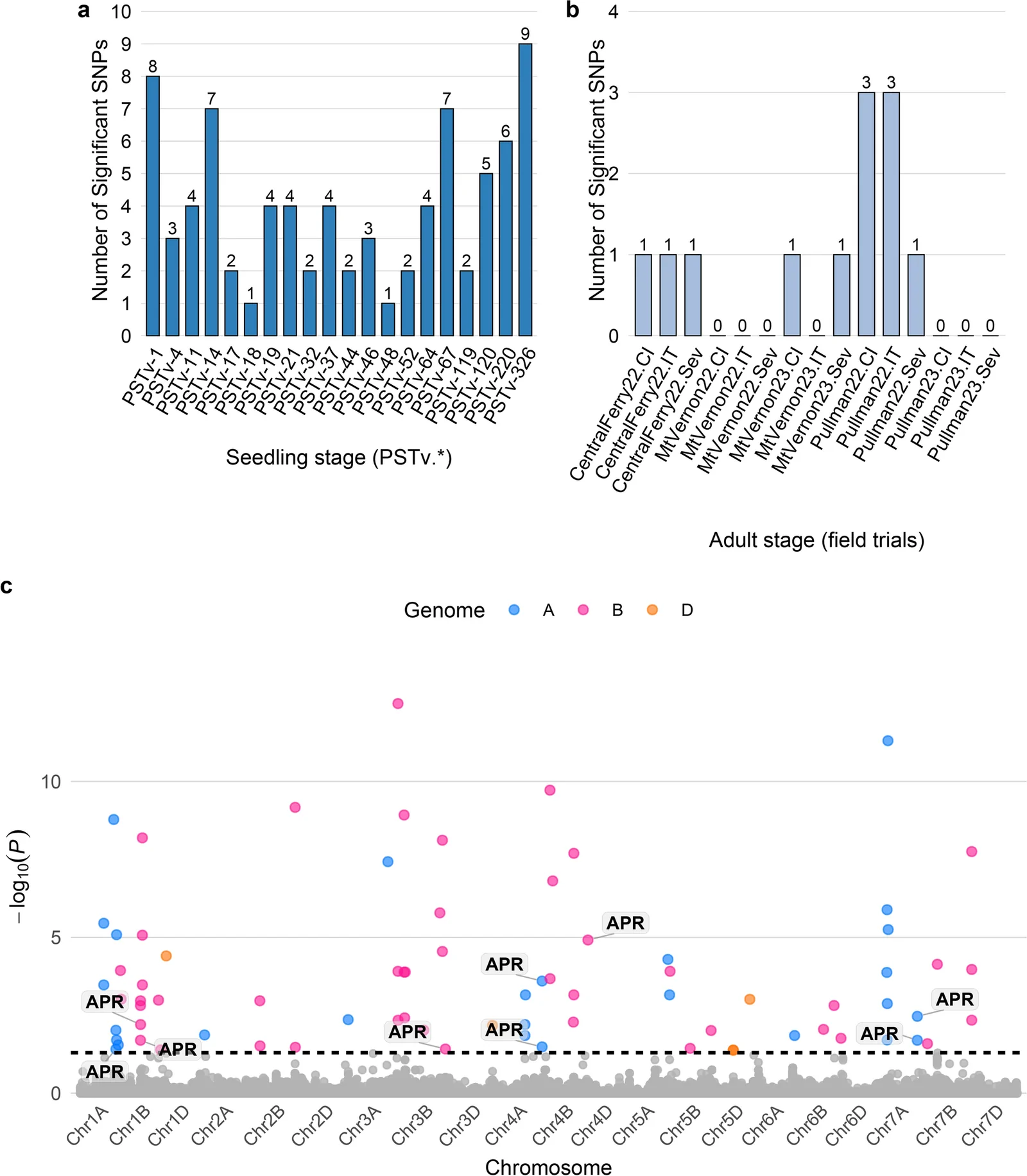
Summary of GWAS results for stripe rust resistance in the greenhouse seedling screens and field trials of adult stage plants. **a** Number of significant SNPs detected in the seedling tests with 20 *Puccinia striiformis* f. sp. *tritici* races. **b** Number of significant SNPs identified in field trials conducted at different locations and scored for coefficient of infection (CI), disease severity (Sev), or infection type (IT). **c** Manhattan plot showing the genomic positions and FDR-corrected significance levels (−log₁₀P) for SNPs associated with resistance across seedling and adult stage trials. SNPs are color-coded by genome (A, B, or D). The dashed line represents the significance threshold (*α* = 0.05). Significant SNPs identified in the field trials at the adult-plant stage are labeled “APR”

Genome-wide association mapping of ASR response to 20 distinct *Pst* isolates revealed 68 non-redundant lead SNPs (Table S8, Fig. 2a and c). These ASR loci were unevenly distributed across the three subgenomes, with the A, B, and D genomes harboring 22, 41, and 5 SNP-trait associations, respectively. Notably, chromosome 3B emerged as a resistance hot spot, harboring 11 SNPs in total, followed by chromosome 1A, which harbored seven significant SNPs. Across the entire GWAS dataset, seven SNPs were significantly associated with IT scores for up to four distinct *Pst* isolates (Table 2), suggesting that certain mapped genomic regions may provide broader-spectrum ASR across the wheat panel. While most SNPs were race-specific and rare, this subset of multi-isolate SNPs may represent promising targets for functional validation and introgression into breeding lines. The number of significant SNPs per race varied widely (Fig. 2b), with PSTv-36 and PSTv-1 contributing the most associations (9 and 8 SNPs, respectively), while several races showed only one or two associations.

**Table 2:** Significant SNPs detected in more than one trial for seedling and adult stage resistance to *Puccinia striiformis* f. sp. *tritici* (*Pst*)

SNP	Number of trials	*Pst* race or location-year of field trials
chr1A_503312428	4	PSTv-17, PSTv-4, PSTv-44, PSTv-46
chr3B_6086348	3	PSTv-19, PSTv-21, PSTv-32
chr3B_673173761	2	PSTv-1, PSTv-220
chr3B_673288150	2	PSTv-1, PSTv-220
chr5D_491107426	3	PST-11, PSTv-120, PSTv-4
chr6B_675439625	2	PSTv-19, PSTv-21
chr7B_190792974	3	PSTv-120, PSTv-37, PSTv-52
chr4A_723639886*	2	CentralFerry22 (CI), CentralFerry22 (Sev)
chr4B_667574481*	3	Pullman 22 (CI), Pullman22 (IT), Pullman22 (Sev)

^*^SNPs associated with resistance in the field trials. CI = coefficient of infection (severity adjusted by reaction type), Sev = disease severity (% leaf area infected), IT = infection type (0–9; lower values indicate higher resistance). Field trial labels combine location, year, and trait.

### GWAS for adult-plant stripe rust responses

The GWAS for stripe rust resistance in the field trials was based on 15 distinct trait–year-environment combinations, including three locations (Pullman, Central Ferry, and Mt. Vernon) and two growing seasons (2022 and 2023). All these locations showed extensive variation in the composition of field *Pst* populations in WA in 2022 and 2023, with each locations including multiple races (https://striperust.wsu.edu/races/data/). The Central Ferry location data from the 2023 field trial were excluded due to low stripe rust pressure (Table S6).

In total, nine non-redundant SNPs were significantly associated with IT, Sev, and CI (Fig. 2b and c, TableS8). These SNPs were distributed among six chromosomes, including 1A, 1B, 3B, 4A, 4B, and 7A (Fig. 2c). The significant SNP detected for IT, CI, and Sev traits on chromosome 4B (chr4B_667574481) was detected only in the Pullman 2022 trial. Likewise, two significant SNPs, one associated with CI and Sev (chr4A_723639886) and another with IT (chr4A_723810464), were detected only in the Central Ferry 2022 trial. The detection of environment-specific SNP associations is likely linked with local variation in the *Pst* race composition or with other location-specific that influenced disease development. No overlap was detected between these nine SNPs and the ASR loci mapped in the greenhouse seedling screens. The association of these SNPs with non-race-specific resistance responses to complex field mixtures of Pst races suggests that they likely represent APR loci.

### Overlap of the mapped loci with the previously reported *Yr* loci

Two approaches have been applied to evaluate the overlap of *Yr* loci mapped in this study with the previously reported *Yr* loci. First, we have used functional KASP markers for five major resistance genes, including *Yr5, Yr15, Yr36, Yr18/Lr34, and Yr29/Lr46*, to genotype the wheat diversity panel (Table S9). Second, a targeted LD-based comparison (*r*2 ≥ 0.6 within ± 10 Mb window) was performed among the 77 *Yr* loci mapped in our study and 1,144 previously reported *Yr* loci (Tong et al. 2024; Wu et al. 2025). Combined, these approaches cover the majority of known ASR and APR *Yr* loci.

The results of KASP genotyping revealed a markedly low occurrence of *Yr5* and *Yr15* in the panel, with *Yr5* being detected in only 12 accessions (3.2%) and *Yr15* missing in the panel (Table S9). These two *Yr* loci confer broad-spectrum ASR and remain effective against all *Pst* races used in this study. The low frequency of *Yr5* and *Yr15* in the panel is consistent with their relatively recent discovery (Marchal et al. 2018; Klymiuk et al. 2021) and targeted introgression into modern elite cultivars, particularly in structured breeding programs, rather than widespread distribution in traditional or landrace germplasm.

Based on KASP genotyping of three APR loci, only *Lr46*/*Yr29* was present at a high frequency, identified in 147 accessions (39%), while *Yr36* was found in just 10 accessions (2.7%). The widely studied slow-rusting gene *Lr34*/*Yr18,* known for conferring durable resistance in many modern cultivars (Krattinger et al. 2009), was completely absent in the panel. The near-complete lack of *Yr36* and *Lr34*/*Yr18* suggests that these APR sources have not been extensively deployed in the geographic or temporal scope represented by the selected lines (Table S9).

**Fig. 3:**
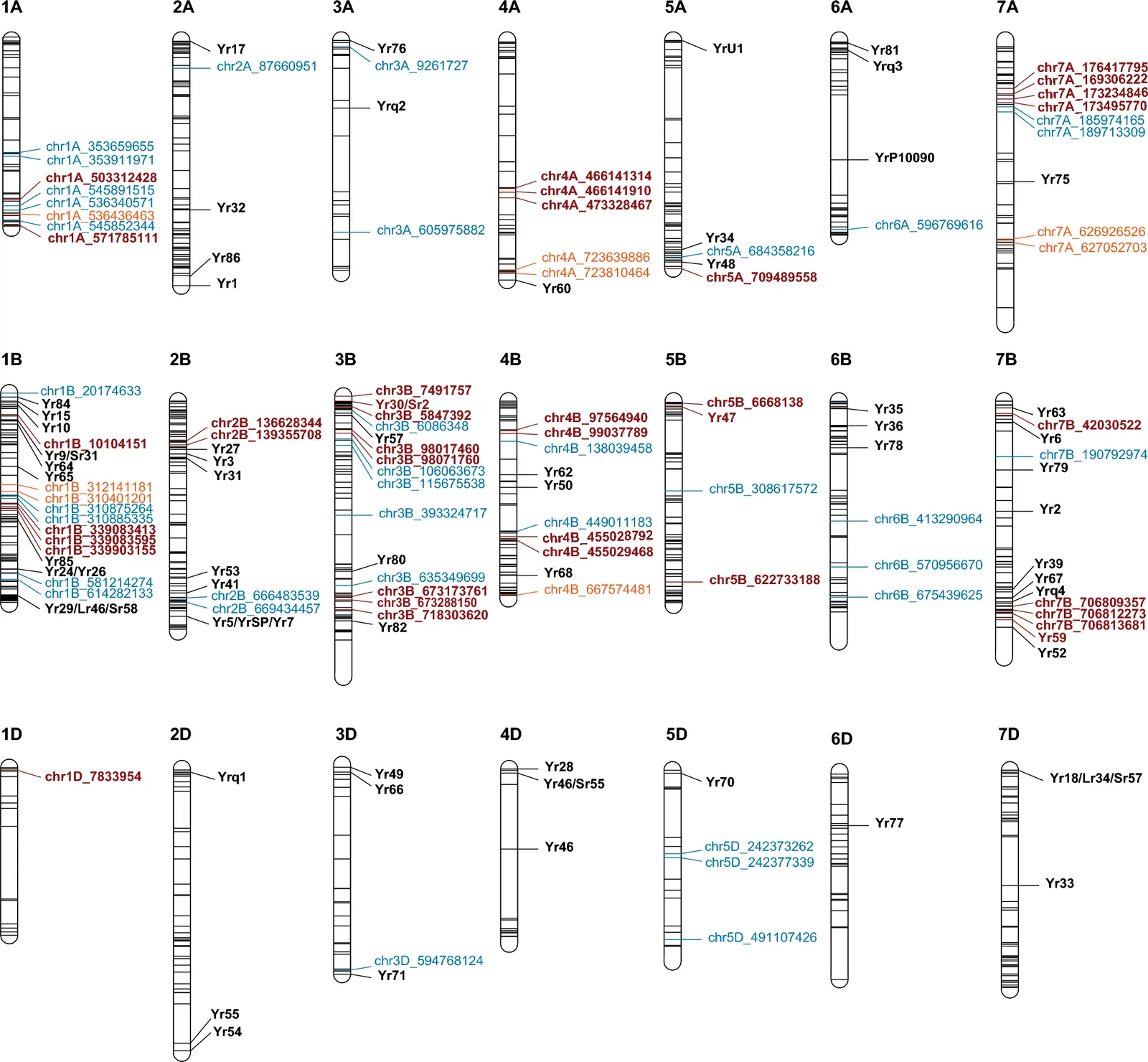
Physical map of wheat *Yr* loci across wheat chromosomes. Black markers show locations of the previously reported Yr genes and QTLs, whereas blue and orange markers show significant SNPs identified in our study in seedling and field evaluations, respectively. Black markers with locus names correspond to known Yr resistance loci that overlap with loci detected in our study. Blue and orange markers with locus names denote novel loci detected in seedling and field evaluations, respectively, that do not coincide with the known Yr loci

A targeted LD-based analyses showed that of the 77 significant SNPs identified in our study, 34 (36%) showed strong LD (*r*2 ≥ 0.6) with 44 known *Yr* genes or QTLs (Fig. 3). Among these, nine SNPs were in perfect LD (*r*2 = 1) with at least one known *Yr* locus. This considerable overlap validates the robustness of our GWAS results and suggests that approximately 36% of detected associations are located within genomic regions harboring previously reported *Yr* loci (Fig. 3; Table S10).

LD-based analysis showed that the race-specific GWAS recovered a substantial fraction of known or previously reported stripe rust resistance regions. Of the 68 non-redundant ASR lead SNPs, 32 co-localized with previously reported *Yr* regions based on physical proximity and LD evidence, and 16 of the 20 race-specific analyses detected at least one SNP overlapping a known resistance region. These overlaps support the biological relevance of the ASR GWAS and indicate that our study captured *Yr* loci consistent with previously characterized stripe rust resistance regions. For example, in the PSTv-64 analysis, chr3B_5847392 co-localized with the *Yr30/Sr2* region (Wang et al. 2024), whereas chr5B_6668138 co-localized with the *Yr47* region (Bansal et al. 2011).

The chromosomal distribution of overlapping loci shows that chromosome 3B harbored the highest number of known resistance loci (n = 13), followed by chromosomes 2B (*n* = 5) and 5B (*n* = 4). Of particular interest, a single region on chromosome 3B displayed a high density of overlapping loci, suggesting the existence of a haplotype block enriched for stripe rust resistance. For example, chr3B_5847392 was found to be in strong LD (*r*2 ~ 0.95) with 13 known resistance loci, including the multi-pathogen resistance gene complex *Sr2*/*Lr27*/*Yr30*, highlighting this locus as a high-value genomic region with potential broad-spectrum effectiveness.

Among the overlapping loci, two well-characterized *Yr* genes, *Yr30* and *Yr47* (Bansal et al. 2011; Wang et al. 2024), were identified within genomic regions exhibiting strong LD with GWAS SNPs. *Yr30*, a component of the *Sr2*/*Lr27*/*Yr30* multi-pathogen resistance locus, was co-localized with chr3B_5847392. Likewise, *Yr47* was detected in perfect LD (*r*2 = 1) with chr5B_6668138, a GWAS-associated SNP also co-localized with three major QTLs: *QYr.eu30*, *QYr.inra-5BL.2*, and *QYr.uga-5B*. The list of best performing lines along with information about the presence of resistant alleles at known *Yr* loci is provided in Table 3. The complete set of resistant alleles mapped in our study in each line from the panel can be found in Table S11.

**Table 3:** Top-performing wheat lines identified from seedling and adult-plant field evaluations for stripe rust resistance

Line	Accession	Origin	Mean seedling IT	Mean field IT	Mean field severity (%)	Known *Yr* marker(s)
GF225	PI 207115	Iran	1.65	2.20	15	None detected
GF244	PI 625725	Iran	1.90	2.00	7	None detected
GF161	PI 189794	Turkey	2.40	1.80	6	None detected
GF231	PI 366981	Afghanistan	2.50	1.80	12	*Yr5*
GF292	PI 638630	Turkey	2.30	2.40	16	None detected
GF35	PI 625506	Iran	1.75	3.40	27	None detected
GF257	PI 137758	Turkey	2.65	3.60	36	None detected
GF104	CItr 15089	Canada	3.75	4.20	38	None detected
GF75*	PI 351907	Switzerland	8.75	3.20	25	*Lr46*/*Yr29*
GF358*	PI 272377	Hungary	8.85	3.40	30	None detected

Mean seedling IT was calculated across the 20 *Puccinia striiformis* f. sp. *tritici* (*Pst*) races evaluated at the seedling stage. Mean field IT and mean field severity were calculated across five adult-plant field environments: Pullman 2022, Mount Vernon 2022, Central Ferry 2022, Pullman 2023, and Mount Vernon 2023. Lower IT and severity values indicate stronger resistance, with IT reflecting infection type and severity reflecting the extent of disease development under field conditions. Known *Yr* marker status was based on diagnostic KASP markers for *Yr5*, *Yr15*, *Yr36*, *Lr46/Yr29*, and *Lr34/Yr18*. ^*^Lines marked with an asterisk showed high mean seedling IT but low mean field IT, consistent with adult-plant resistance expressed under field conditions.

We detected one APR locus mapped in our study to chromosome 3B (chr3B_718303620) that overlapped with the previously mapped stripe rust resistance locus *QYr.agis104* (Cheng et al. 2024). There was also one SNP associated with APR in our panel, chr1B_614282133, that mapped 47 Mb away from *Yr29*/*Lr46* (William et al. 2003), which is located at 661 Mb on chromosome 1B. We also detected a significant GWAS SNP on chromosome 7B (chr7B_4706809357) that was in strong LD with the APR locus *Yr59* (Table S10). However, this GWAS SNP was detected in the race-specific seedling resistance screens rather than in the field-based trials (Table S8). Therefore, this SNP likely colocalizes with the *Yr59* genomic region rather than directly tagging the *Yr59* locus itself. Combined, our results suggest that the majority of loci detected in our study show little overlap with the genomic regions previously associated with APR.

### Wheat improvement had distinct impact on ASR and APR alleles

**Fig. 4:**
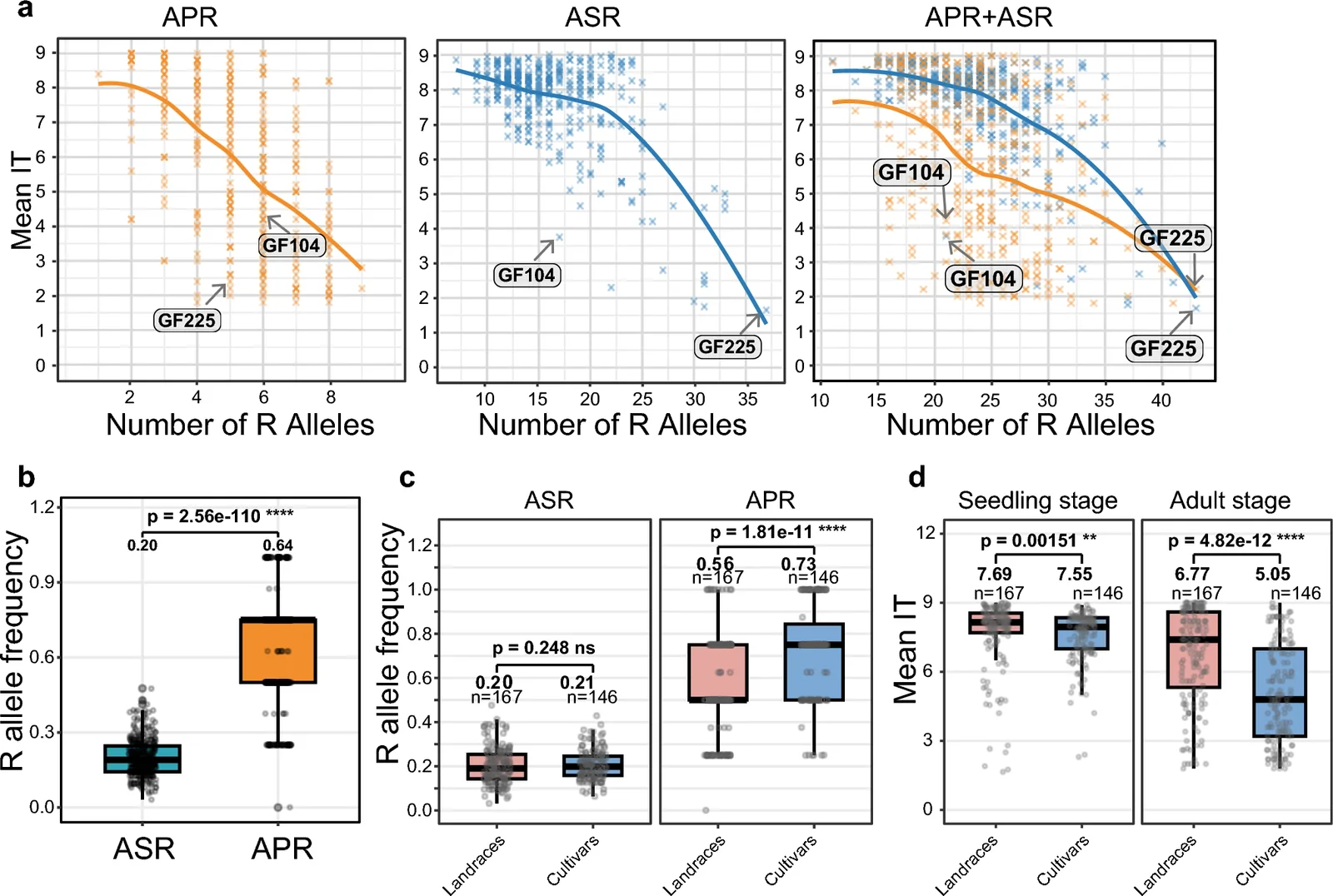
Distribution of resistance (*R*) gene loci across populations and among wheat lines. **a** The X-axis shows the number of *R* alleles per line. Y-axis shows the mean infection-type (IT) scores based on data from 20 *Pst* races for ASR and field infection trials for APR. Both adult-plant and seedling stage screening data were combined for assessing the impact of total number of APR and ASR R alleles on IT. The data collected at the adult and seedling stages are shown as orange and blue crosses, respectively. **b** Comparison of R allele frequencies between ASR and APR loci. A nonparametric Mann–Whitney U-test was applied to compare allele frequencies. **c** Comparison of ASR and APR allele frequencies between landraces and cultivars. Within each growth stage, group differences were tested using a one-sided Mann–Whitney U-test. **d** Comparison of IT collected at the seedling and adult-plant stages between landraces and cultivars. Data were grouped into landraces and cultivars (cultivars and breeding selections), excluding genetic resources. Mean IT values and sample sizes (n) are indicated above each box, and horizontal brackets denote p values from one-sided Mann–Whitney U-test. Significance levels: **** *p* < 0.0001; *** *p* < 0.001; ** *p* < 0.01; * *p* < 0.05; and ns = not significant

Pyramiding ASR and APR genes increases resistance to wheat rusts (Zhang et al. 2019; Wang et al. 2023; Wu et al. 2025). While our results, in general, are consistent with these observations for *Yr* loci detected in both seedling and adult stage screens (Fig. 4a, Table S11), we also found that the phenotypic effects of APR and ASR accumulation have distinct dynamic. Increase in the number of ASR alleles had little effect on mean IT of the majority of wheat lines in the panel (Fig. 4a). This is likely associated with the fact that the majority of *Yr* genes mapped in the seedling screens are race-specific and have limited contribution to mean IT calculated from data obtained using 20 *Pst* races. Only limited fraction of wheat lines showed mean IT ≤ 6 (Fig. 4a, Table S11), including GF225 and GF104 lines, which were resistant against all 20 tested *Pst* isolates. Contrary to ASR loci, combinations of 4–5 APR alleles in many wheat lines were sufficient to provide good levels of resistance (IT ≤ 6) in the field trials. The threefold higher frequency of resistant alleles at APR loci compared to those at ASR loci (Mann–Whitney U-test *p* value = 2.6 × 10^−110^) could have also facilitated accumulation of multiple APR alleles in some wheat lines (Fig. 4b).

The differences in the accumulation of resistant alleles between APR and ASR loci could be associated with differences in the historic selection that acted on these loci. Selection for ASR, which are mostly represented by race-specific *Yr* genes, varies across time and space and largely driven by “boom and bust cycles” of wheat–*Pst* interaction. The *Yr* loci detected in field trials under natural infection conditions, which include mixes of *Pst* isolates varying from year-to-year in composition, likely include few all-stage race-specific resistance genes and mostly APR loci that have more quantitative non-race-specific effects. If this assumption is correct, we may expect that selection associated with wheat improvement preferentially targeted APR alleles rather than ASR alleles. To test this hypothesis, we compared the changes in resistant allele frequency for APR and ASR loci between landraces and more modern improved wheat cultivars, including breeding material and cultivars. Consistent with our assumption, we observed no changes in resistant allele frequency for AST between landraces and cultivars (one-sided Mann–Whitney U-test, *p* value = 0.25), whereas resistant alleles at APR loci showed 30.3% increase in cultivars compared to landraces (one-sided Mann–Whitney U-test, *p* value = 1.8 × 10^−11^) (Fig. 4c). The changes in IT mirrored the results of resistant allele frequency comparison; transition from landraces to cultivars was associated with only 1.8% reduction in IT for seedling stage resistance (one-sided Mann–Whitney U-test, p value = 1.5 × 10^−3^), whereas IT at adult stage reduced by 25.5% (one-sided Mann–Whitney U-test, *p* value = 4.8 × 10^−12^).

## Discussion

The continued evolution of *Pst* has rendered many resistance genes deployed in wheat cultivars ineffective, increasing the urgency to identify novel and durable sources of resistance (Wan and Chen 2014; Schwessinger 2016; Shahinnia et al. 2022; Riella et al. 2024; Davis et al. 2025). This study used a globally representative panel of wheat lines, including landraces, historical cultivars, and breeding lines, to evaluate resistance to stripe rust. The panel spans more than a century of wheat development and captures geographic and genetic diversity from key centers of domestication and adaptation (He et al. 2022). This region-specific selection led to accumulation of a broad spectrum of resistance alleles, including those that are underrepresented in modern breeding programs (Adhikari et al. 2012; Zegeye et al. 2014). Using a diverse set of 20 *Pst* isolates, we discovered a wide range of infection types, revealing considerable variation in resistance responses and the potential to uncover both established and novel resistance loci.

ASR genes effective against multiple stripe rust isolates, especially *Yr5* and *Yr15*, are broadly used in developing resistant wheat varieties (Yaniv et al. 2015; Klymiuk et al. 2018; Marchal et al. 2018) and represent valuable targets for gene discovery efforts. Five lines from our diversity panel (GF104, GF161, GF225, GF23, and GF257) showed resistance to 18 or more of the 20 *Pst* races, including the highly virulent races PSTv-37, PSTv-52, PSTv-220, and PSTv-326. Among these lines, GF104 (CItr 15089) and GF225 (PI 207115) were resistant to all 20 races, with both lines being resistant at the adult-plant stage too. Diagnostic marker analysis revealed that none of the lines in our diversity panel carried well-characterized ASR genes *Yr5* (Marchal et al. 2018) and *Yr15* (Klymiuk et al. 2018), indicating that other, potentially novel or under-characterized ASR loci may contribute to the observed resistance in GF104 and GF225. In light of recent reports that resistance conferred by Yr5 and Yr15 has been broken (Tekin et al. 2021; Davis et al. 2025), identifying wheat lines that remain resistant to a broad range of Pst races provides potentially novel sources of ASR genes for breeding durable stripe rust resistance.

Among the APR genes screened using KASP markers in our panel, three well-characterized loci, *Yr29*/*Lr46*, *Yr18*/*Lr34*, and *Yr36* (William et al. 2003; Lagudah et al. 2009; Fu et al. 2009), were specifically targeted due to their roles in providing durable, non-race-specific resistance to stripe rust. These genes function through partial resistance mechanisms and are often more effective when combined with other *Yr* genes, showing additive effects in gene pyramiding strategies (Singh et al. 2016; Wang et al. 2023). They are also associated with pleiotropic resistance to multiple pathogens, including leaf, stem, and stripe rust (Krattinger et al. 2009; Lagudah 2011). In our panel, only *Yr29*/*Lr46* was present at high frequency (39%), while *Yr36* was present at low frequency (2.7%) and *Yr18*/*Lr34* was not detected. The lack of overlap between the *Yr* loci detected in our study and these major APR genes indicate that variation in resistance at the adult-plant stage is largely driven by alternative, potentially novel sources of APR in the panel. For example, two lines in the panel, GF75 and GF358, were susceptible to all 20 *Pst* races at the seedling stage but consistently exhibited resistance or intermediate resistance across all adult-plant field trials, which were dominated by *Pst* race PSTv-37 virulent on 97.3% of wheat lines in the greenhouse screens. GF75 tested positive for *Yr29*/*Lr46*, while GF358 lacked all three APR markers screened in this study (*Yr18*/*Lr34*, *Yr29*/*Lr46*, and *Yr36*). These examples confirm that lines in our diversity panel harbor the APR sources that have yet to be fully characterized.

In spite of extensive prior mapping efforts focused on discovery of the *Yr* resistance genes in diverse germplasm (Tong et al. 2024; Wu et al. 2025), almost 60% of the *Yr* loci mapped in our study showed no linkage with the previously mapped genes using the given thresholds. We also cannot rule out the possibility that *Yr* loci showing strong linkage with the previously mapped resistance genes are novel loci until higher resolution genetic mapping studies are performed. Some previous GWAS studies, particularly those utilizing narrower genetic panels or fewer pathogen races, reported higher co-localization rates with known resistance loci (Maccaferri et al. 2015; Shahinnia et al. 2022). This relatively lower match rate may reflect the broader genetic diversity and pathogen variation encompassed in our panels of wheat lines and *Pst* isolates, leading to the discovery of potentially novel and previously uncharacterized associations.

One of the interesting aspects of our data are the significant differences in population R allele frequency observed for *Yr* genes detected in the seedling and adult-plant screens. These differences are likely associated with the differences in the expression of disease resistance traits controlled by the mapped *Yr* genes. The ASR *Yr* genes are usually associated with race-specific strong dominant resistance expressed at all stages of development and could be easily selected during breeding process (Chen 2013; Wang et al. 2023). The strong phenotypic effects expressed by this class of resistance genes also apply strong selection pressure on pathogen populations leading to the origin of new races that overcome resistance (Brown 2015; Riella et al. 2024). The shifts in the composition of pathogen populations refocus local breeding efforts on other strong effect resistance genes that confer resistance against newly emerged pathogen races. As a result, most ASR genes never reach high frequency in diverse wheat panels, which is consistent with our data. On the contrary, many APR *Yr* genes express moderate-to-weak race non-specific resistance and likely impose weaker selection pressure on pathogen populations, preventing quick evolution of new virulent races. At the times when major resistance genes became non-effective due to evolution of virulent races, it is likely that selection would favor genotypes that carry the race non-specific genes with weaker effects. In addition, these loci potentially enhance resistance when combined with ASR genes (Zhang et al. 2019; Wang et al. 2023). Both factors can contribute to consistent long-term selection pressure on quantitative disease resistance loci and drive their frequency to higher levels in more modern germplasm, as was observed in our diversity panel.

In conclusion, this study demonstrates the value of continued screening of diverse global germplasm using diverse collections of pathogen races for discovering novel sources of resistance for breeding. Variation in the representation of ASR and APR loci in various diversity panels combined with extensive variation at avirulence loci in the populations of pathogens (Olivera et al. 2019; Guo et al. 2022; Riella et al. 2024) offer unique opportunities for uncovering the genetic makeup of interaction between wheat and its pathogens and identifying novel loci to protect wheat production from disease outbreaks. Using a panel composed of landraces, historical cultivars, and breeding lines, we identified 77 *Yr* loci conferring ASR or APR. A total of 44 *Yr* loci were potentially novel sources of resistance that warrant further investigation. Importantly, we identify two accessions, GF104 (CItr 15089) and GF225 (PI 207115), that exhibited resistance to all 20 *Pst* races yet lacked diagnostic markers for the broadly effective ASR genes *Yr5* and *Yr15*, suggesting that their resistance is conferred by previously uncharacterized ASR alleles. Overall, these findings not only confirm the value of known *Yr* loci but also expand the catalog of candidate loci available for marker-assisted selection. Together, our results provide actionable targets for diversifying the genetic base of resistance and advancing the development of wheat cultivars with broad-spectrum, durable resistance against evolving *Pst* populations.

## Supplementary Information

Below is the link to the electronic supplementary material.

Supplementary file1 (XLSX 188 KB)

Supplementary file2 (PDF 4255 KB)

Supplementary file3 (PDF 28 KB)

Supplementary file4 (DOCX 16 KB)

## Data Availability

Bio Sample IDs of wheat lines are linked with NCBI accession numbers PRJNA787276 and PRJNA1480388. The genotyping data used in this study have been deposited in the European Variation Archive (EVA) at EMBL-EBI under accession number PRJNA1480388. Phenotyping data collected in this study are provided as supplementary information. The germplasm is available through the USDA-ARS Germplasm Resources Information Network (GRIN).

## References

[CR1] Adhikari T, Gurung S, Hansen J et al (2012) Association mapping of quantitative trait loci in spring wheat landraces conferring resistance to bacterial leaf streak and spot blotch. Plant Genome 5:1–16

[CR2] Ballini E, Lauter N, Wise R (2013) Prospects for advancing defense to cereal rusts through genetical genomics. Front Plant Sci 4:117. 10.3389/fpls.2013.00117 PMC3640194 23641250

[CR3] Bansal UK, Forrest KL, Hayden MJ et al (2011) Characterisation of a new stripe rust resistance gene Yr47 and its genetic association with the leaf rust resistance gene Lr52. Theor Appl Genet 122:1461–1466 10.1007/s00122-011-1545-4 21344185

[CR4] Beddow JM, Pardey PG, Chai Y et al (2015) Research investment implications of shifts in the global geography of wheat stripe rust. Nat Plants 1:15132. 10.1038/nplants.2015.132 27251389

[CR5] Benjamini Y, Hochberg Y (1995) Controlling the false discovery rate: a practical and powerful approach to multiple testing. J R Stat Soc Ser B Stat Methodol 57:289–300

[CR6] Brown JKM (2015) Durable resistance of crops to disease: a Darwinian perspective. Annu Rev Phytopathol 53:513–539. 10.1146/annurev-phyto-102313-045914 26077539

[CR7] Browning BL, Tian X, Zhou Y, Browning SR (2021) Fast two-stage phasing of large-scale sequence data. Am J Hum Genet 108:1880–1890. 10.1016/j.ajhg.2021.08.005 PMC8551421 34478634

[CR8] Bulli P, Zhang J, Chao S et al (2016) Genetic architecture of resistance to stripe rust in a global winter wheat germplasm collection. G3 (Bethesda) 6:2237–2253. 10.1534/g3.116.028407 PMC4978880 27226168

[CR9] Cao Q, Zhu Z, Xu D et al (2024) Characterization of a 4.1 Mb inversion harboring the stripe rust resistance gene YR86 on wheat chromosome 2AL. Crop J 12:1168–1175. 10.1016/j.cj.2024.05.011

[CR10] Chen XM (2005) Epidemiology and control of stripe rust [*Puccinia striiformis* f. sp. *tritici*] on wheat. Can J Plant Pathol 27:314–337. 10.1080/07060660509507230

[CR11] Chen X (2013) High-temperature adult-plant resistance, key for sustainable control of stripe rust. Am J Plant Sci 04:608–627. 10.4236/ajps.2013.43080

[CR12] Chen X (2020) Pathogens which threaten food security: *Puccinia striiformis*, the wheat stripe rust pathogen. Food Secur 12:239–251. 10.1007/s12571-020-01016-z

[CR13] Cheng S, Feng C, Wingen LU et al (2024) Harnessing landrace diversity empowers wheat breeding. Nature 632:823–831. 10.1038/s41586-024-07682-9 PMC11338829 38885696

[CR14] Chhetri M, Bariana H, Kandiah P, Bansal U (2016) Yr58: A new stripe rust resistance gene and its interaction with Yr46 for enhanced resistance. Phytopathology 106:1530–1534. 10.1094/PHYTO-04-16-0182-R 27673348

[CR15] Cleveland WS (1979) Robust locally weighted regression and smoothing scatterplots. J Am Stat Assoc 74:829–836. 10.1080/01621459.1979.10481038

[CR68] Chen X, Wang M (2025) Race characterization of Puccinia striiformis f. sp. tritici and P. striiformis f. sp. hordei in the United States. Springer, New York 10.1007/978-1-0716-4378-5_2 40198548

[CR16] Covarrubias-Pazaran G (2016) Genome-assisted prediction of quantitative traits using the r package sommer. PLoS ONE 11:1–15. 10.1371/journal.pone.0156744 PMC4894563 27271781

[CR17] Davis HR, Chia T, Rhodes H et al (2025) First large-scale breakdown of Yr15 resistance to wheat yellow rust (*Puccinia striiformis* f. sp. *tritici*) recorded globally. New Dis Reports 52:2–5. 10.1002/ndr2.70067

[CR18] Du X, Hu L, Xu W et al (2025) Mapping, validation, and development of markers for the major adult plant stripe rust resistance locus QYr.hnk-7DL in Henan wheat. BMC Plant Biol. 10.1186/s12870-025-06918-8 PMC12220352 40604473

[CR69] FAOSTAT (2023) Crops and Livestock Products. Food and Agriculture Organization of the United Nations. https://www.fao.org/faostat/en/#home

[CR19] Fu D, Uauy C, Distelfeld A et al (2009) A kinase-START gene confers temperature-dependent resistance to wheat stripe rust. Science 323:1357–1360. 10.1126/science.1166289 PMC4737487 19228999

[CR20] Gao Z, Wang X, Li Y et al (2024) Evaluation of stripe rust resistance and genome-wide association study in wheat varieties derived from the International Center for Agricultural Research in the Dry Areas. Front Plant Sci 15:1–10. 10.3389/fpls.2024.1377253 PMC11035757 38654905

[CR21] Guo Y, Betzen B, Salcedo A et al (2022) Population genomics of *Puccinia graminis* f.sp. *tritici* highlights the role of admixture in the origin of virulent wheat rust races. Nat Commun 13:1–16. 10.1038/s41467-022-34050-w PMC9587050 36271077

[CR22] He F, Wang W, Rutter WB et al (2022) Genomic variants affecting homoeologous gene expression dosage contribute to agronomic trait variation in allopolyploid wheat. Nat Commun 13:826. 10.1038/s41467-022-28453-y PMC8837796 35149708

[CR23] Hill WG, Robertson A (1968) Linkage disequilibrium in finite populations. Theor Appl Genet 38:226–231. 10.1007/BF01245622 24442307

[CR24] Hovmøller MS, Walter S, Bayles RA et al (2016) Replacement of the European wheat yellow rust population by new races from the centre of diversity in the near-Himalayan region. Plant Pathol 65:402–411. 10.1111/ppa.12433

[CR25] Huerta-Espino J, Singh R, Crespo-Herrera LA, Villaseñor-Mir HE, Rodriguez-Garcia MF, Dreisigacker S, Barcenas-Santana D, Lagudah E (2020) Adult Plant Slow Rusting Genes Confer High Levels of Resistance to Rusts in Bread Wheat Cultivars From Mexico Front. Plant Sci 11:824 10.3389/fpls.2020.00824 PMC7371971 32760411

[CR70] Jia M, Yang L, Zhang W et al (2020) Genome-wide association analysis of stripe rust resistance in modern Chinese wheat. BMC Plant Biol 20:1–13. 10.1186/s12870-020-02693-w PMC7590722 33109074

[CR26] Kertho A, Mamidi S, Bonman JM et al (2015) Genome-wide association mapping for resistance to leaf and stripe rust in winter-habit hexaploid wheat landraces. PLoS ONE 10:7–9. 10.1371/journal.pone.0129580 PMC4468153 26076040

[CR27] Klymiuk V, Yaniv E, Huang L et al (2018) Cloning of the wheat Yr15 resistance gene sheds light on the plant tandem kinase-pseudokinase family. Nat Commun. 10.1038/s41467-018-06138-9 PMC6170490 30282993

[CR28] Klymiuk V, Coaker G, Fahima T, Pozniak CJ (2021) Tandem protein kinases emerge as new regulators of plant immunity. Mol Plant-Microbe Interact 34:1094–1102. 10.1094/MPMI-03-21-0073-CR PMC8761531 34096764

[CR29] Krattinger SG, Lagudah ES, Spielmeyer W et al (2009) A putative ABC transporter confers durable resistance to multiple fungal pathogens in wheat. Science 323:1360–1363. 10.1126/science.1166453 19229000

[CR30] Lagudah ES (2011) Molecular genetics of race non-specific rust resistance in wheat. Euphytica 179:81–91. 10.1007/s10681-010-0336-3

[CR31] Lagudah ES, Krattinger SG, Herrera-Foessel S et al (2009) Gene-specific markers for the wheat gene Lr34/Yr18/Pm38 which confers resistance to multiple fungal pathogens. Theor Appl Genet 119:889–898. 10.1007/s00122-009-1097-z 19578829

[CR32] Li Y, Lin R, Hu J et al (2022) Mapping of wheat stripe rust resistance gene Yr041133 by BSR-Seq analysis. Crop J 10:447–455. 10.1016/j.cj.2021.06.009

[CR33] Line RF, Qayoum A (1992) Virulence, aggressiveness, evolution, and distribution of races of Puccinia striiformis (the cause of stripe rust of wheat) in North America 1968-87, U.S Department of Agriculture Technical Bulletin the National Technical Information Service 44 1788

[CR34] Liu L, Wang M, Zhang Z et al (2020) Identification of stripe rust resistance loci in U.S. spring wheat cultivars and breeding lines using genome-wide association mapping and Yr gene markers. Plant Dis 104:2181–2192. 10.1094/PDIS-11-19-2402-RE 32511046

[CR71] Lopes MS, El-Basyoni I, Baenziger PS et al (2015) Exploiting genetic diversity from landraces in wheat breeding for adaptation to climate change. J Exp Bot 66:3477–3486. 10.1093/jxb/erv122 25821073

[CR35] Maccaferri M, Zhang J, Bulli P et al (2015) A genome-wide association study of resistance to stripe rust (*Puccinia striiformis* f. sp. *tritici*) in a worldwide collection of hexaploid spring wheat (*Triticum aestivum* L.). G3 (Bethesda) 5:449–465. 10.1534/g3.114.014563 PMC4349098 25609748

[CR36] Marchal C, Zhang J, Zhang P et al (2018) BED-domain-containing immune receptors confer diverse resistance spectra to yellow rust. Nat Plants 4:662–668. 10.1038/s41477-018-0236-4 30150615

[CR37] McIntosh RA (2024) Catalogue of Gene Symbols for Wheat. In: GrainGenes A Database Triticeae Avena

[CR38] Nsabiyera V, Bariana HS, Qureshi N et al (2018) Characterisation and mapping of adult plant stripe rust resistance in wheat accession Aus27284. Theor Appl Genet 131:1459–1467. 10.1007/s00122-018-3090-x 29560515

[CR39] Olivera PD, Sikharulidze Z, Dumbadze R et al (2019) Presence of a sexual population of *Puccinia graminis* f. sp. *tritici* in Georgia provides a hotspot for genotypic and phenotypic diversity. Phytopathology 109:2152–2160. 10.1094/PHYTO-06-19-0186-R 31339468

[CR40] Peterson RF, Campbell AB, Hannah AE (1948) A diagrammatic scale for estimating rust intensity on leaves and stems of cereals. Can J Res 26c:496–500. 10.1139/cjr48c-033

[CR41] Purcell S, Neale B, Todd-Brown K et al (2007) PLINK: a tool set for whole-genome association and population-based linkage analyses. Am J Hum Genet 81:559–575. 10.1086/519795 PMC1950838 17701901

[CR42] Rasheed A, Wen W, Gao F et al (2016) Development and validation of KASP assays for genes underpinning key economic traits in bread wheat. Theor Appl Genet 129:1843–1860. 10.1007/s00122-016-2743-x 27306516

[CR43] Riella V, Rodriguez-Algaba J, García R et al (2024) New races with wider virulence indicate rapid evolution of *Puccinia striiformis* f. sp. *tritici* in the Southern Cone of America. Plant Dis 108:2454–2461. 10.1094/PDIS-02-24-0320-RE 38537139

[CR72] Reif JC, Zhang P, Dreisigacker S et al (2005) Wheat genetic diversity trends during domestication and breeding. Theor Appl Genet 110:859–864. 10.1007/s00122-004-1881-8 15690175

[CR44] Saari EE, Wilcoxson R (2003) Plant disease situation of high-yielding dwarf wheats in Asia and Africa. Annu Rev Phytopathol 12:49–68. 10.1146/annurev.py.12.090174.000405

[CR45] Schwessinger B (2016) Tansley insight Fundamental wheat stripe rust research in the 21st century. New Phytol 213(1–7):1625–1631 10.1111/nph.14159 27575735

[CR46] Segura V, Vilhjálmsson BJ, Platt A et al (2012) An efficient multi-locus mixed-model approach for genome-wide association studies in structured populations. Nat Genet 44:825–830. 10.1038/ng.2314 PMC3386481 22706313

[CR73] Sehgal D, Dreisigacker S, Belen S et al (2016) Mining centuries old in situ conserved Turkish wheat landraces for grain yield and stripe rust resistance genes. Front Genet 7:1–15. 10.3389/fgene.2016.00201 PMC5114521 27917192

[CR47] Shahinnia F, Geyer M, Schürmann F et al (2022) Genome-wide association study and genomic prediction of resistance to stripe rust in current Central and Northern European winter wheat germplasm. Theor Appl Genet 135:3583–3595. 10.1007/s00122-022-04202-z PMC9519682 36018343

[CR48] Singh RP, Hodson DP, Jin Y et al (2015) Emergence and spread of new races of wheat stem rust fungus: continued threat to food security and prospects of genetic control. Phytopathology 105:872–884. 10.1094/PHYTO-01-15-0030-FI 26120730

[CR49] Singh RP, Singh PK, Rutkoski J et al (2016) Disease impact on wheat yield potential and prospects of genetic control. Annu Rev Phytopathol 54:303–322. 10.1146/annurev-phyto-080615-095835 27296137

[CR50] Tekin M, Cat A, Akan K et al (2021) A new virulent race of wheat stripe rust pathogen (*Puccinia striiformis* f. sp. *tritici*) on the resistance gene Yr5 in Turkey. Plant Dis 105:3292

[CR51] The International Wheat Genome Sequencing Consortium (IWGSC) (2018) Shifting the limits in wheat research and breeding using a fully annotated reference genome. Science 361:eaar7191. 10.1126/science.aar7191 30115783

[CR52] Tong J, Zhao C, Liu D et al (2024) Genome-wide atlas of rust resistance loci in wheat. Theor Appl Genet 137:1–16. 10.1007/s00122-024-04689-8 PMC11233289 38980436

[CR53] VanRaden PM (2008) Efficient methods to compute genomic predictions. J Dairy Sci 91:4414–4423. 10.3168/jds.2007-0980 18946147

[CR54] Wan A, Chen X (2014) Virulence characterization of *Puccinia striiformis* f. sp. *tritici* using a new set of Yr single-gene line differentials in the United States in 2010. Plant Dis 98:1534–1542. 10.1094/PDIS-01-14-0071-RE 30699782

[CR55] Wang J, Zhang Z (2021) GAPIT version 3: boosting power and accuracy for genomic association and prediction. Genomics Proteomics Bioinforma 19:629–640. 10.1016/j.gpb.2021.08.005 PMC9121400 34492338

[CR56] Wang M, Wan A, Chen X (2022) Race characterization of *Puccinia striiformis* f. sp. *tritici* in the United States from 2013 to 2017. Plant Dis 106:1462–1473 10.1094/PDIS-11-21-2499-RE 35077227

[CR57] Wang F, Zhang M, Hu Y et al (2023) Pyramiding of adult-plant resistance genes enhances all-stage resistance to wheat stripe rust. Plant Dis 107:879–885. 10.1094/PDIS-07-22-1716-RE 36044366

[CR58] Wang X, Xiang M, Li H et al (2024) High-density mapping of durable and broad-spectrum stripe rust resistance gene Yr30 in wheat. Theor Appl Genet 137:1–13. 10.1007/s00122-024-04654-5 38850423

[CR59] Wickham H (2016) ggplot2: Elegant Graphics for Data Analysis. Springer-Verlag 10:978

[CR60] William M, Singh RP, Huerta-Espino J et al (2003) Molecular marker mapping of leaf rust resistance gene Lr46 and its association with stripe rust resistance gene Yr29 in wheat. Phytopathology 93:153–159. 10.1094/PHYTO.2003.93.2.153 18943129

[CR61] WSU (2022) Washington State University (WSU) Annual Stripe Rust Race Data Reports. Washington State University

[CR62] Wu J, Ma S, Niu J et al (2025) Genomics-driven discovery of superior alleles and genes for yellow rust resistance in wheat. Nat Genet 57:2017–2027. 10.1038/s41588-025-02259-2 40696174

[CR63] Yaniv E, Raats D, Ronin Y et al (2015) Evaluation of marker-assisted selection for the stripe rust resistance gene Yr15, introgressed from wild emmer wheat. Mol Breed. 10.1007/s11032-015-0238-0 PMC5091809 27818611

[CR74] Yu J, Pressoir G, Briggs WH et al (2006) A unified mixed-model method for association mapping that accounts for multiple levels of relatedness. Nat Genet 38:203–208. 10.1038/ng1702 16380716

[CR64] Zegeye H, Rasheed A, Makdis F et al (2014) Genome-wide association mapping for seedling and adult plant resistance to stripe rust in synthetic hexaploid wheat. PLoS ONE 9:e105593. 10.1371/journal.pone.0105593 PMC4143293 25153126

[CR65] Zhang B, Chi D, Hiebert C et al (2019) Pyramiding stem rust resistance genes to race TTKSK (Ug99) in wheat. Can J Plant Pathol 41:443–449. 10.1080/07060661.2019.1596983

[CR66] Zheng X, Zhou J, Zhang M et al (2022) Transfer of durable stripe rust resistance gene Yr39 into four Chinese elite wheat cultivars using marker-assisted selection. Agronomy (Basel) 12:1–12. 10.3390/agronomy12081791

[CR67] Zhu T, Wang L, Rimbert H et al (2021) Optical maps refine the bread wheat *Triticum aestivum* cv. Chinese Spring genome assembly. Plant J 107:303–314. 10.1111/tpj.15289 PMC8360199 33893684

[CR75] Zuev EV, Lebedeva TV, Yakovleva OV et al (2024) Genetic Diversity for Effective Resistance in Wheat Landraces from Ethiopia and Eritrea to Fungal Diseases and Toxic AluminumIons. Plants 13:1166. 10.3390/plants13081166 PMC11053688 38674575

