# Supplementary material for: Genetic architecture and evolution of stripe rust resistance uncovered using diverse panels of wheat lines and North American Puccinia striiformis f. sp. tritici isolates
Source: Theor Appl Genet. 2026 Sep 8;139(10):263. doi: 10.1007/s00122-026-05363-x (PMC13553620; doi:10.1007/s00122-026-05363-x)
Supplement: Supplementary file 1 [file 122_2026_5363_MOESM2_ESM.pdf]

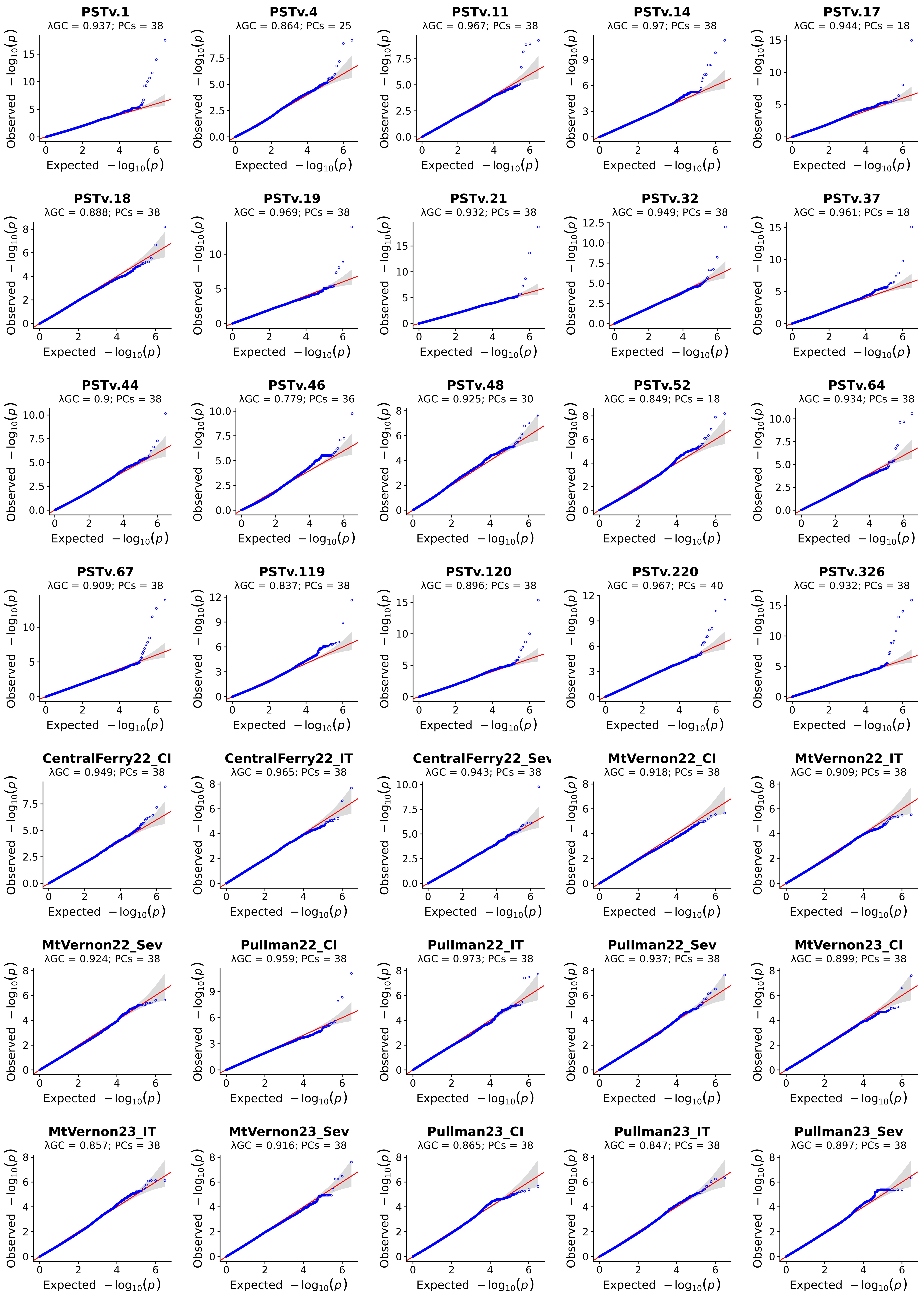

PSTv.1
λGC = 0.937; PCs = 38
PSTv.4
λGC = 0.864; PCs = 25
PSTv.11
λGC = 0.967; PCs = 38
PSTv.14
λGC = 0.97; PCs = 38
PSTv.17
λGC = 0.944; PCs = 18
PSTv.18
λGC = 0.888; PCs = 38
PSTv.19
λGC = 0.969; PCs = 38
PSTv.21
λGC = 0.932; PCs = 38
PSTv.32
λGC = 0.949; PCs = 38
PSTv.37
λGC = 0.961; PCs = 18
PSTv.44
λGC = 0.9; PCs = 38
PSTv.46
λGC = 0.779; PCs = 36
PSTv.48
λGC = 0.925; PCs = 30
PSTv.52
λGC = 0.849; PCs = 18
PSTv.64
λGC = 0.934; PCs = 38
PSTv.67
λGC = 0.909; PCs = 38
PSTv.119
λGC = 0.837; PCs = 38
PSTv.120
λGC = 0.896; PCs = 38
PSTv.220
λGC = 0.967; PCs = 40
PSTv.326
λGC = 0.932; PCs = 38
CentralFerry22_CI
λGC = 0.949; PCs = 38
CentralFerry22_IT
λGC = 0.965; PCs = 38
CentralFerry22_Sev
λGC = 0.943; PCs = 38
MtVernon22_CI
λGC = 0.918; PCs = 38
MtVernon22_IT
λGC = 0.909; PCs = 38
MtVernon22_Sev
λGC = 0.924; PCs = 38
Pullman22_CI
λGC = 0.959; PCs = 38
Pullman22_IT
λGC = 0.973; PCs = 38
Pullman22_Sev
λGC = 0.937; PCs = 38
MtVernon23_CI
λGC = 0.899; PCs = 38
MtVernon23_IT
λGC = 0.857; PCs = 38
MtVernon23_Sev
λGC = 0.916; PCs = 38
Pullman23_CI
λGC = 0.865; PCs = 38
Pullman23_IT
λGC = 0.847; PCs = 38
Pullman23_Sev
λGC = 0.897; PCs = 38
Observed $-\log_{10}(p)$
Expected $-\log_{10}(p)$
