# Supplementary material for: Genetic architecture and evolution of stripe rust resistance uncovered using diverse panels of wheat lines and North American Puccinia striiformis f. sp. tritici isolates
Source: Theor Appl Genet. 2026 Sep 8;139(10):263. doi: 10.1007/s00122-026-05363-x (PMC13553620; doi:10.1007/s00122-026-05363-x)
Supplement: Supplementary file 2 [file 122_2026_5363_MOESM3_ESM.pdf]

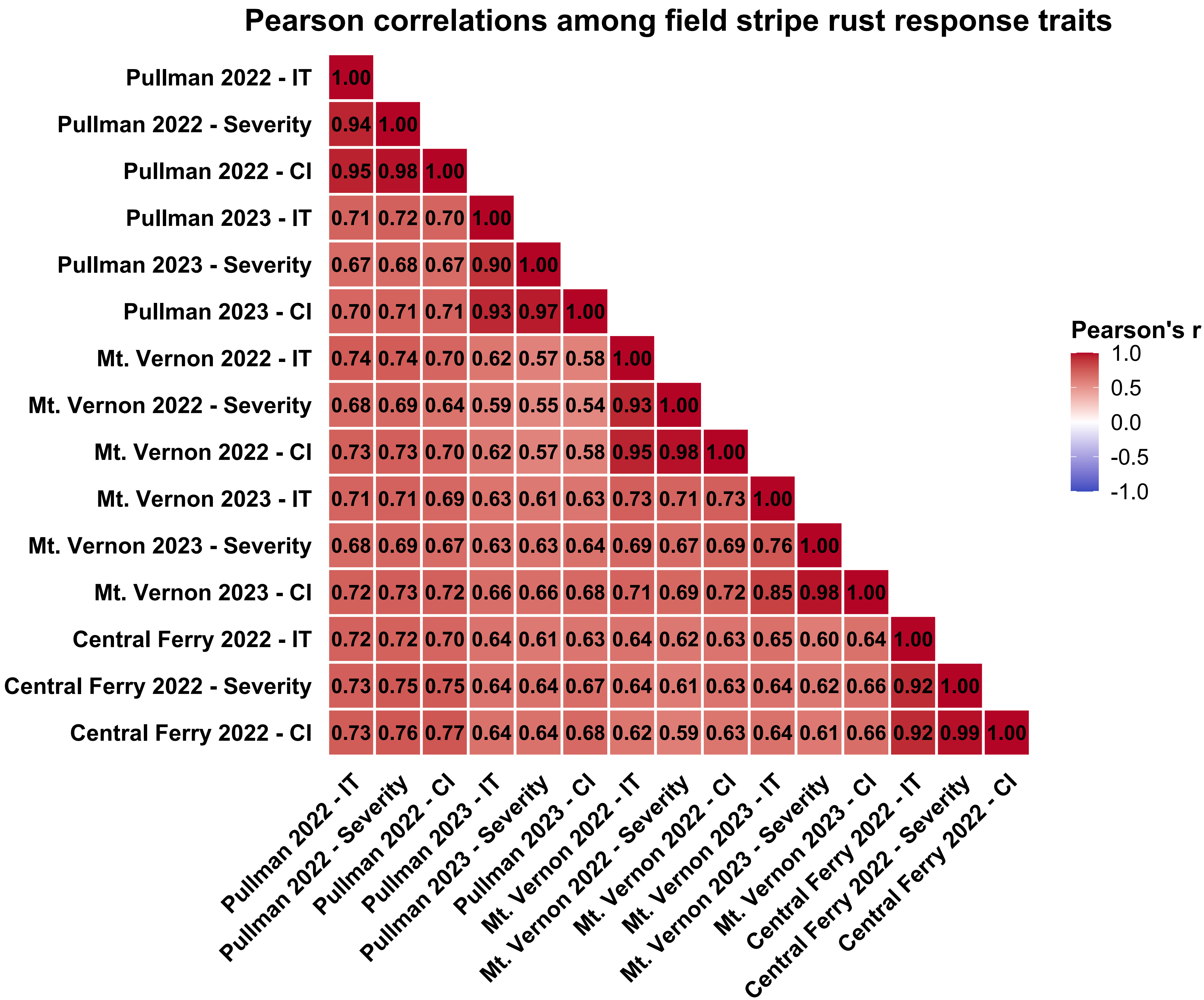
Pearson correlations among field stripe rust response traits
Pullman 2022 - IT 1.00
Pullman 2022 - Severity 0.94 1.00
Pullman 2022 - CI 0.95 0.98 1.00
Pullman 2023 - IT 0.71 0.72 0.70 1.00
Pullman 2023 - Severity 0.67 0.68 0.67 0.90 1.00
Pullman 2023 - CI 0.70 0.71 0.71 0.93 0.97 1.00
Mt. Vernon 2022 - IT 0.74 0.74 0.70 0.62 0.57 0.58 1.00
Mt. Vernon 2022 - Severity 0.68 0.69 0.64 0.59 0.55 0.54 0.93 1.00
Mt. Vernon 2022 - CI 0.73 0.73 0.70 0.62 0.57 0.58 0.95 0.98 1.00
Mt. Vernon 2023 - IT 0.71 0.71 0.69 0.63 0.61 0.63 0.73 0.71 0.73 1.00
Mt. Vernon 2023 - Severity 0.68 0.69 0.67 0.63 0.63 0.64 0.69 0.67 0.69 0.76 1.00
Mt. Vernon 2023 - CI 0.72 0.73 0.72 0.66 0.66 0.68 0.71 0.69 0.72 0.85 0.98 1.00
Central Ferry 2022 - IT 0.72 0.72 0.70 0.64 0.61 0.63 0.64 0.62 0.63 0.65 0.60 0.64 1.00
Central Ferry 2022 - Severity 0.73 0.75 0.75 0.64 0.64 0.67 0.64 0.61 0.63 0.64 0.62 0.66 0.92 1.00
Central Ferry 2022 - CI 0.73 0.76 0.77 0.64 0.64 0.68 0.62 0.59 0.63 0.64 0.61 0.66 0.92 0.99 1.00
Pullman 2022 - IT
Pullman 2022 - Severity
Pullman 2022 - CI
Pullman 2023 - IT
Pullman 2023 - Severity
Pullman 2023 - CI
Mt. Vernon 2022 - IT
Mt. Vernon 2022 - Severity
Mt. Vernon 2022 - CI
Mt. Vernon 2023 - IT
Mt. Vernon 2023 - Severity
Mt. Vernon 2023 - CI
Central Ferry 2022 - IT
Central Ferry 2022 - Severity
Central Ferry 2022 - CI
Pearson's r
1.0
0.5
0.0
-0.5
-1.0
