# Supplementary material for: Genetic architecture and evolution of stripe rust resistance uncovered using diverse panels of wheat lines and North American Puccinia striiformis f. sp. tritici isolates
Source: Theor Appl Genet. 2026 Sep 8;139(10):263. doi: 10.1007/s00122-026-05363-x (PMC13553620; doi:10.1007/s00122-026-05363-x)
Supplement: Supplementary file 3 [file 122_2026_5363_MOESM4_ESM.docx]

**Supplementary Tables**

**Table S1.** Passport information for 377 spring wheat accessions, including line names, plant IDs, improvement status (landrace or cultivar), region of origin, and year of collection.

**Table S2.** *Pst* race composition summarized by site and year for adult-plant stripe rust resistance field trials.

**Table S3.** Summary of PC selection and genomic inflation statistics for seedling *Pst* races and adult-plant field phenotypes.

**Table S4.** Seedling infection type scores for 377 wheat lines evaluated against 20 *Puccinia striiformis* f. sp. *tritici* (*Pst*) races under controlled conditions. Includes avirulence counts and corresponding percentages per line.

**Table S5.** Field-based stripe rust evaluations conducted in 2022 across three environments (Pullman, Mount Vernon, and Central Ferry, WA). Severity (%), infection type (IT), and coefficient of infection (CI) are reported for each line per location.

**Table S6.** Stripe rust resistance data from 2023 field trials conducted in Pullman and Mount Vernon, WA. Severity (%), infection type (IT), and coefficient of infection (CI) are reported for each line per location.

**Table S7.** Descriptive statistics (mean, standard deviation, range, and quartiles) for 35 phenotypic traits, including seedling infection types across 20 *Puccinia striiformis* f. sp. *tritici* races and field-based severity, infection type, and coefficient of infection.

**Table S8.** Summary of significant SNPs associated with stripe rust resistance identified through genome-wide association study (GWAS) with Multi-locus Mixed Model (MLMM).

**Table S9.** Genotyping results for 377 spring wheat accesions using five diagnostic KASP markers linked to known stripe rust resistance genes. The table indicates the presence ("Yes") or absence ("No") of resistance-associated alleles for *Yr15*, *Yr5*, *Yr36*, *Lr46*/*Yr29*, and *Lr34*/*Yr18* in each line.

**Table S10.** Pairwise linkage disequilibrium (LD) between significant GWAS SNPs and nearby SNPs within ±10 Mb, based on the IWGSC RefSeq v1.0 genome. For each GWAS-identified SNP, the table lists the chromosome, physical position, and SNP ID, along with the corresponding LD-linked SNP, its genomic position, LD value (r² ≥ 0.6), and any overlapping previously reported resistance gene or QTL.

**Table S11.** Distribution of resistant alleles of GWAS SNP markers across the GF wheat panel.

**Supplementary Figures**

**Figure S1.** Quantile–quantile (Q–Q) plots comparing observed and expected p-values for each trait included in the GWAS. The optimal number of PCs and genomic inflation factor for each trait are shown on the top of each plot.

**Figure S2.** Pairwise Pearson’s correlation coefficients (*r*) were calculated among adult-plant stripe rust response traits measured across field environments using the ‘cor’ function in R v4.2.1. The analysis included infection type (IT), disease severity (Sev), and coefficient of infection (CI) scores from Pullman 2022, Pullman 2023, Mount Vernon 2022, Mount Vernon 2023, and Central Ferry 2022. Correlations were calculated using pairwise complete observations and visualized as a lower-triangle heatmap using the ‘ggplot2’ package in R.
